# What Azure blues occur in Canada? A re-assessment of *Celastrina* Tutt species (Lepidoptera, Lycaenidae)

**DOI:** 10.3897/zookeys.584.7882

**Published:** 2016-04-26

**Authors:** B. Christian Schmidt, Ross A. Layberry

**Affiliations:** 1 Canadian National Collection of Insects, Arachnids and Nematodes, Agriculture and Agri-Food Canada, K.W. Neatby Bldg., 960 Carling Ave., Ottawa, ON, Canada K1A 0C6; 2 6124 Carp Road, Kinburn, Ontario, Canada K0A 2H0

**Keywords:** Voltinism, Cornus, Viburnum, Eastern Flowering Dogwood, Eriophyidae, Cherry gall, degree-day model, DNA barcode

## Abstract

The identity of *Celastrina* species in eastern Canada is reviewed based on larval host plants, phenology, adult phenotypes, mtDNA barcodes and re-assessment of published data. The status of the Cherry Gall Azure (*Celastrina serotina* Pavulaan & Wright) as a distinct species in Canada is not supported by any dataset, and is removed from the Canadian fauna. Previous records of this taxon are re-identified as *Celastrina lucia* (Kirby) and *Celastrina neglecta* (Edwards). Evidence is presented that both *Celastrina lucia* and *Celastrina neglecta* have a second, summer-flying generation in parts of Canada. The summer generation of *Celastrina lucia* has previously been misidentified as *Celastrina neglecta*, which differs in phenology, adult phenotype and larval hosts from summer *Celastrina lucia*. DNA barcodes are highly conserved among at least three North American *Celastrina* species, and provide no taxonomic information. *Celastrina neglecta* has a Canadian distribution restricted to southern Ontario, Manitoba, Saskatchewan and easternmost Alberta. The discovery of museum specimens of *Celastrina ladon* (Cramer) from southernmost Ontario represents a new species for the Canadian butterfly fauna, which is in need of conservation status assessment.

## Introduction

Blues of the genus *Celastrina* Tutt, commonly known as azures, are perhaps the most familiar spring butterflies in Canada, occurring in all ecoregions except the high arctic. Despite their ubiquity, their identification and taxonomy is difficult, with species boundaries and nomenclature having a long history of controversy and confusion. Forty years ago, all North American *Celastrina* taxa were generally considered to represent variation within a single species described from Europe, *Celastrina argiolus* (L.) (Langston 1975). This view remained essentially unchanged for another twenty years, with the exception of a second taxon, *Celastrina nigra* (Forbes) recognized by Miller and Brown (1981) and Scott (1986). A global revision of *Celastrina* and related genera further entrenched the concept of only two North American species (Eliot and Kawazoé 1983). However, with a more detailed study of the genus in North America additional cryptic species were gradually recognized by some (Opler and Krizek 1984, Pratt et al. 1994, Scott and Wright 1998, Wright and Pavulaan 1999, Pavulaan and Wright 2005). *Celastrina* taxonomy is still unsettled, with recent comprehensive North American checklists varying between three (NABA 2001) and nine recognized species (Pelham 2008). A summary of some of the changing concepts, particularly in the historical literature, is given by Pratt et al. (1994) and Pavulaan (2014).

The conservative morphological variation between most *Celastrina* species, coupled with adult seasonal polyphenism, has been a major impediment to *Celastrina* taxonomy and dictated a gradual refinement of species concepts. Comparative data on molecular variation, physiology, development and ecology for sympatric or closely parapatric populations are therefore particularly important in evaluating species concepts, yet such data are largely lacking (but see Pavulaan 2014). To provide a taxonomic reference point for Canada’s *Celastrina* populations and to stimulate further study, the identity of Ontario *Celastrina* populations is re-assessed based on published and novel data on phenology, larval host plant use and mtDNA variation. Ontario provides a unique geographic arena where biological and biogeographical attributes of putative species can be examined. Here, three species purportedly occur in sympatry: *Celastrina lucia* (Kirby), *Celastrina serotina* Pavulaan & Wright and *Celastrina neglecta* (Edwards) (Layberry et al. 1998, Pavulaan and Wright 2005). A fourth species, *Celastrina ladon* (Cramer), has been reported from adjacent parts of Ohio and Michigan (Nielsen 1999). With potentially as many as four species present in Ontario, life history traits and diagnostic characters of *Celastrina* were studied and compared among two ecoregions, the Lake Erie region in southernmost Ontario and the Ottawa region in eastern Ontario. These regions were chosen as both have a long history of entomology with a comparatively large data pool on Lepidoptera, and represent separate ecoregions with all three (and potentially four) eastern Canadian *Celastrina* species present.

### Current concepts of eastern Canadian *Celastrina*

Four *Celastrina* species are currently attributed to the Canadian fauna, three of them found in the East. The fourth species, *Celastrina echo* (Edwards), is strictly western and although previously ranked as a subspecies of *Celastrina ladon* (Cramer) (e.g. Layberry et al. 1998), it is now recognized as a distinct species by most authors (e.g. Guppy and Shepard 2001, Pohl et al. 2009, Warren 2005, James and Nunnallee 2011, CESCC 2011). The concept of three eastern Canadian species as presented by Layberry et al. (1998) is in current usage (Hall et al. 2014, eButterfly 2015, Macnaughton et al. 2015), with some nomenclatural updates (Table 1).

**Table 1.** Changing concepts of Canadian *Celastrina* species.

Current concept (Pelham 2011)	Pavulaan and Wright 2005	NABA 2001	Wright and Pavulaan 1999	Layberry et al. 1998	Pratt et al. 1994	Scott 1984
*Celastrina lucia*	*lucia* (+ *lucia* auct.)	*Celastrina ladon*	*Celastrina ladon lucia*	*Celastrina ladon lucia*	*Celastrina ladon lucia*	*Celastrina argiolus*
*Celastrina serotina*	*Celastrina serotina*	*Celastrina ladon*	*Celastrina ladon ladon*	*Celastrina* sp. n.	*Celastrina ladon* “violacea II”	*Celastrina argiolus*
*Celastrina neglecta*	*Celastrina neglecta*	*C ladonneglecta*	*Celastrina neglecta*	*Celastrina neglecta*	*Celastrina ladon neglecta*	*Celastrina argiolus*
*Celastrina echo*	n/a	*Celastrina ladon*	n/a	*Celastrina ladon nigrescens*, *Celastrina ladon echo*	*Celastrina ladon nigrescens*, *Celastrina ladon echo*	*Celastrina argiolus*
*Celastrina ladon*	*Celastrina ladon*	*Celastrina ladon*	*Celastrina ladon ladon*	n/a	*Celastrina ladon* “violacea I”	*Celastrina argiolus*

*Celastrina lucia*, the Northern Azure (a.k.a. Spring Azure, a name here reserved for *Celastrina ladon*), is the most widespread azure, occurring in every province and territory. In the boreal and subarctic regions it is the only species of the genus. The Northern Azure has been considered to be univoltine throughout its range, flying in early spring (Layberry et al. 1998, Pavulaan 2014). Populations south of the boreal region, where adults are slightly larger and with a more variable ventral wing pattern, have been treated as a separate taxon (*Celastrina* “*lucia*” of authors), also considered to be a univoltine spring-flying species (Pratt et al. 1994, Pavulaan 2014). There is currently no available scientific name for this taxon, nor is it clear that one is needed, as it may merely represent ecophenotypic variation of boreal *Celastrina lucia*. Larvae of *Celastrina lucia* feed on a wide variety of flowering shrubs but, like *Celastrina serotina*, occasionally also on cherry galls in some parts of the range (Pavulaan 2014).

*Celastrina neglecta*, the Summer Azure, has a more southerly but overlapping distribution with *Celastrina lucia* and is recorded from all provinces except British Columbia, Newfoundland and Labrador. It is distinguished from *Celastrina lucia* by its later flight season, in Canada flying mostly in July, six to eight weeks after the peak flight of spring-flying *Celastrina lucia*. All summer-flying *Celastrina* in southern Canada have been assigned to *Celastrina neglecta* (Layberry et al. 1998, Hall et al. 2014, eButterfly 2015), based on the premise that *Celastrina lucia* is univoltine, and that the time between spring (*Celastrina lucia*) and summer (*Celastrina neglecta*) *Celastrina* flights is not enough for a summer flight to represent a second generation of *Celastrina lucia* (noted as early as Saunders 1875). However, Eberlie (1996, 1997) documented that late-summer larvae from Northumberland County (Ontario), by definition *Celastrina neglecta*, can produce typical early-spring *Celastrina lucia* adults the following year. This phenomenon has also been documented in the Ottawa region (Layberry 2004). The diagnostic value of phenology is complicated further by the possibility that *Celastrina neglecta* sometimes has an earlier flying, spring brood according to Pavulaan (2014), which is difficult or impossible to segregate morphologically from *Celastrina lucia*. Conversely, the possibility of second-generation *Celastrina lucia* has not been adequately evaluated in Canadian populations.

*Celastrina serotina*, the Cherry Gall Azure, is also a univoltine species but with a late spring flight, between that of *Celastrina lucia* and *Celastrina neglecta*. There is some doubt in the species status of Ontario populations of *Celastrina serotina*, as larvae reared from cherry galls in the spring can produce *Celastrina neglecta*-type adults in the same season (Layberry 2004). The peak flight time of *Celastrina serotina* is from late May to late June in Ontario, about three weeks after that of *Celastrina lucia*, and before the *Celastrina neglecta* peak in July (Pavulaan and Wright 2005). The larvae are said to feed almost exclusively on eriophyid mite galls on black cherry (*Prunus serotina* Ehrh.) and choke cherry (*Prunus virginiana* L.) leaves. The phenology and larval host plant are key diagnostic features used to distinguish *Celastrina serotina*. Also, the ventral hindwing pattern is stated to be paler whitish grey on average than *Celastrina lucia*, with heavily marked forms being rare. The taxonomy of Ontario *Celastrina serotina* is particularly relevant since life histories and specimens of these populations formed part of the original species description (Wright and Pavulaan 2005). *Celastrina serotina* has also been reported from Québec, New Brunswick, Nova Scotia and Prince Edward Island (Layberry et al. 1998), and recently from Manitoba (based on larval collections, leg. T. Rapati; eButterfly 2015, record #EB-3473).

**Figure 1.**
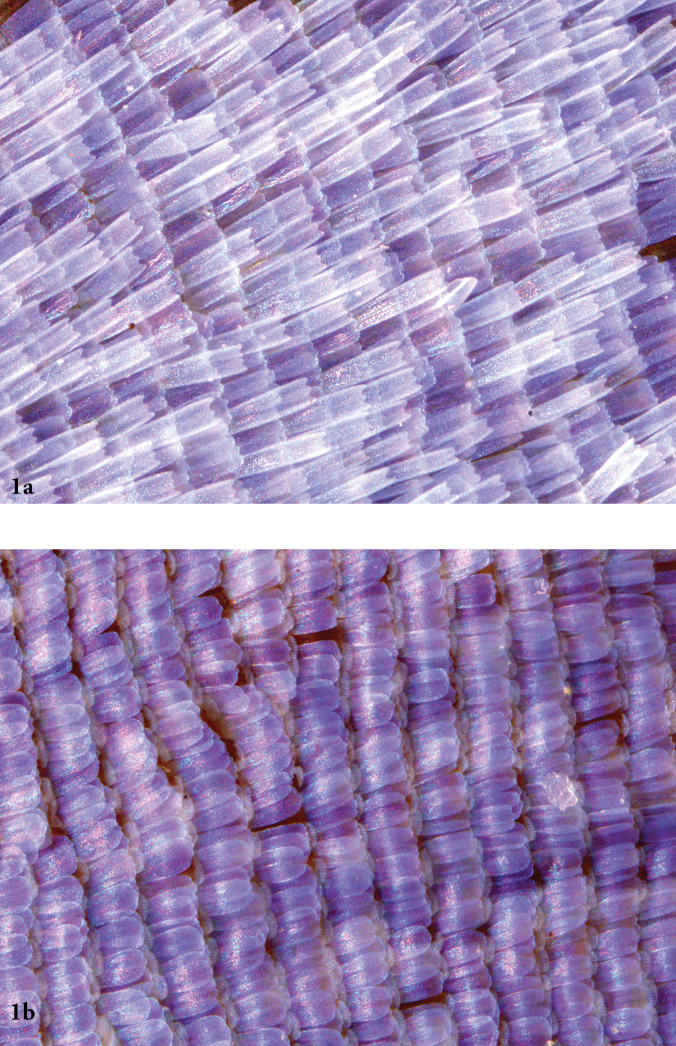
**a** Male *Celastrina ladon* forewing showing distinctive overlapping scales and lack of androconial scales. Normandale, ON. **b** Male *Celastrina lucia* forewing showing pale, underlying androconial scales typical of this species and *Celastrina neglecta*.

*Celastrina ladon*, the Spring Azure, has not been reported in Canada in the sense of the modern concept of the species, where the diagnostic male wing scale morphology (Fig. 1) separates it from all other *Celastrina* (see also Omura 2015; Wright and Pavulaan 1999). Literature reports of *Celastrina ladon* in Canada consist of previous concepts where *Celastrina ladon* and *Celastrina lucia* were considered to be conspecific (e.g. Wright and Pavulaan 1999). *Celastrina ladon* has subsequently not been included in the Canadian fauna (Hall et al. 2014). Older reports of *Celastrina ladon* from southern Ontario may have included true *Celastrina ladon*, but these records cannot be distinguished from *Celastrina lucia* without voucher material. The Spring Azure is known from adjacent parts of southern Michigan (Nielsen 1999) and Ohio (Wright 1998).

## Methods and materials

Specimens examined during this study included those deposited in the Canadian National Collection of Insects, Arachnids and Nematodes (CNC), in addition to *Celastrina* records with voucher photographs on eButterfly (2015). Forewing androconial scales of male *Celastrina* were examined using a Leica 205C dissecting scope. Vouchers of reared specimens are deposited in the CNC.

### DNA barcodes

Molecular variation of *Celastrina* species was assessed using the COI barcode fragment, with DNA extraction, PCR amplification, and sequencing performed at the Canadian Centre for DNA Barcoding (CCDB), following standard protocols (CCDB 2013). Public barcode sequence records were available for three North American species (*Celastrina lucia*, *Celastrina echo* and *Celastrina neglecta*), and the Eurasian *Celastrina argiolus* and Asian *Celastrina morsheadi* (Evans). Novel sequences were generated for 31 eastern Ontario specimens (Suppl. material 1), initially identified as *Celastrina neglecta* (five wild-collected specimens and five reared from larvae collected in late July to August), *Celastrina serotina* (10 specimens reared from larvae feeding on cherry galls in mid June), and *Celastrina lucia* (four specimens collected in May).

DNA sequences were analyzed on the Barcode of Life Data Systems website (BOLD, www.boldsystems.org). The dataset was filtered to include only records with sequences greater than 600 base-pairs in length, and with voucher specimen photographs and collection data that made independent species identification possible. Sequence variation was analyzed using the Kimura-2-Parameter (K2P) distance model and the neighbor-joining (NJ) algorithm as implemented on BOLD. Voucher specimen data is given in Suppl. material 1.

### Larval development and host plants

Larvae were collected from the wild to compare phenology and voltinism of *Celastrina lucia* and *Celastrina serotina*, and to obtain comparative study specimens unambiguously associated with the current concept of *Celastrina serotina*. *Celastrina serotina* is univoltine with a peak flight after that of *Celastrina lucia* (Pavulaan and Wright 2005), so larvae develop later in the season with the resulting pupae entering diapause until the following spring. Larval sampling was carried out in eastern Ontario (Table 2) by directed visual searches and the use of a beating sheet. Numerous species of flowering shrubs were sampled, with most effort directed to sampling *Cornus*, *Viburnum* and *Prunus*. Larvae were reared indoors under natural light:dark conditions and at a constant 20 °C, reflecting the June mean daily temperature of 20.4 °C for Ottawa (Environment and Climate Change Canada 2015).

**Table 2.** Locality data for study sites mentioned in text.

site #	Locality	Lat	Long
1	CAN: ON, Ottawa, Stony Swamp Conservation Area, Richmond Rd.	45.29	-75.83
2	CAN: ON, Ottawa, Stony Swamp Conservation Area, Timm Dr.	45.315	-75.86
3	CAN: ON, Ottawa, Stony Swamp Conservation Area, Cassidy Rd.	45.323	-75.806
4	CAN: ON, Ottawa, Stony Swamp Conservation Area, Watts Ck.	45.341	-75.869
5	CAN: ON, Ottawa-Carleton Dist., Carp Hills	45.386	-76.075
6	CAN: ON, Hastings Co., Madoc, 3km W	44.5	-77.51
7	CAN: ON, Lanark Co., Pakenham, 4 km W, 9th Concession Rd.	45.304	-76.331
8	CAN: ON, Lanark Co., Pakenham, 12 km SW, Bellamy Rd.	45.276	-76.418

### Flight phenology

As a proxy for mean seasonal abundances of *Celastrina* taxa, observation records spanning from 1895-2014 were compiled from the Ontario Butterfly Atlas (Macnaughton et al. 2015). Each unique location-date record was treated as one observation event, regardless of *Celastrina* abundance during that event. Observation frequency (abundance) by date was assessed for two ecoregions, the Great Lakes-St. Lawrence Mixed Forest of easternmost Ontario and the Carolinian Forest of southernmost Ontario (Scott 1995). The southern Ontario dataset included 1056 records from Brantford, Elgin, Essex, Kent, Lambton, Middlesex, Niagara and Norfolk counties; eastern Ontario data consisted of 2145 records from Ottawa-Carleton, Lanark, Russell, Prescott, Glengarry, Stormont and Dundas counties. Three *Celastrina* taxa were considered to occur in each region, but to avoid *a priori* assumptions about species identities, all *Celastrina* records were combined.

Assessing flight peaks based on phenological data combined for multiple taxa could underestimate the number of taxa, if relative abundance discrepancies are large and flights overlap. Emergence patterns were therefore independently assessed through field surveys of eggs, larvae and adults 1–2 × per week in 2015. These data were supplemented with *Celastrina* records and accompanying voucher photographs available on eButterfly (2015).

To assess between-region differences in adult emergence times due to climatic differences, phenology data were examined using a simple degree-day model (e.g. Kelker et al. 1990; Dearborn and Westwood 2014) using the formula:

DD_LTT_ = [((T_max_ - T_min_) / 2)–LTT]

where DD = degree-days, T_max_ and T_min_ = daily maximum and minimum temperatures, respectively, and LTT = the lower threshold temperature of insect development. LTT is the temperature at which physiological development is negligible, for the species and life stage under study. LTT values of 6 °C to 10 °C are generally implemented for insects, with values in the lower range corresponding to temperate-zone species (e.g. Kelker et al. 1990). As *Celastrina* are cold-adapted and some of the first lepidopterans to emerge from winter-diapausing pupae, LTT was set at 6 °Celastrina A start date of April 1^st^ was chosen as DD accumulation values were zero prior to this date (for all values of LTT between 6 °C and 10 °C). Daily temperature data were obtained for 2009–2015 for two stations, Ottawa (city station) for the eastern Ontario region, and London for the southern Ontario region (Environment Canada 2015). A few instances of missing daily maximum/minimum temperature data were estimated by averaging the corresponding temperature from the preceding and following day. Daily maximum and minimum temperatures were calculated based on a six-year average from 2009–2015. London was chosen as representative of the Lake Erie region as it is inland from the Lake Erie shoreline and therefore less prone to cooling climatic effects of onshore winds along the immediate shoreline region.

## Results and discussion

### Wing Scale structure

Examination of forewing scale structure in male *Celastrina* specimens from southern and eastern Ontario led to the discovery of four specimens of *Celastrina ladon*: ON, [Norfolk Co.], Normandale, 22.May.1956, J.R. Lonsway; ON, [Norfolk Co.], St. Williams, 7.May.1977, J.T. Troubridge; ON, Elgin Co., Calton Swamp WMA, 7.May.2000, I. Carmichael. Two female specimens are likely also *Celastrina ladon*, one from Normandale, 28.May.1956, J.R. Lonsway, and one from St. Williams with the same date and collector as the male. All are from the Carolinian forest region of Lake Erie (Fig. 6). *Celastrina ladon* is therefore confirmed as part of the Canadian fauna for the first time. Although other literature and even photo records may exist, voucher specimens are needed to verify identification, at least until phenotypic variation and distribution of *Celastrina ladon* in southern Ontario is better documented. Unvouchered previous records are of little value in ascertaining *Celastrina* identities in southern Ontario, underscoring the importance of voucher study specimens even when a species is thought to be common and well-known.

### DNA barcodes

**Figure 2.**
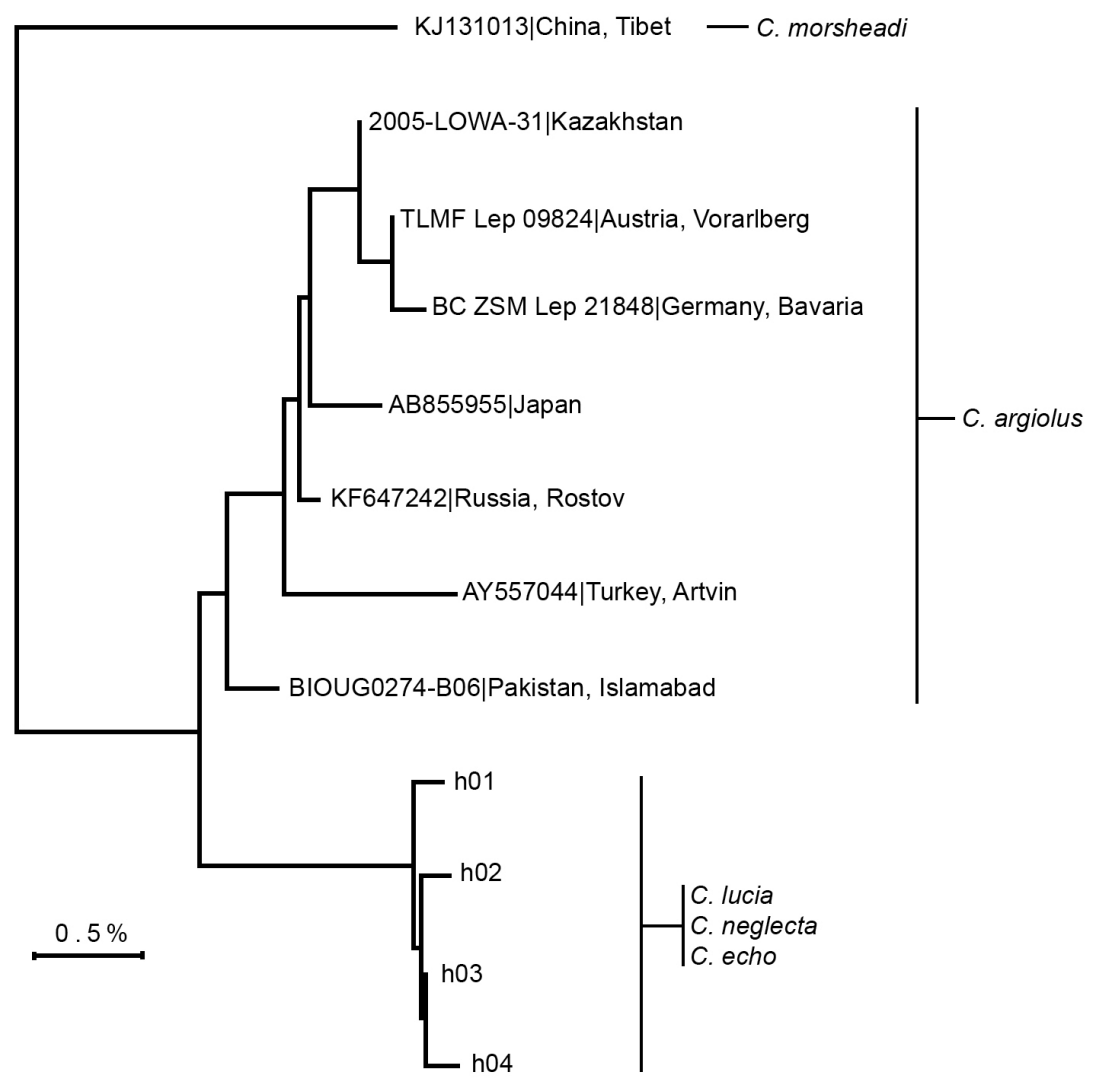
Neighbour-joining tree of DNA barcode sequences for *Celastrina*, with specimen voucher number and country of origin at branch tips. North American samples include 79 samples represented by four haplotypes, with h03 shared among three species (n=76) and remaining three haplotypes with one sample each of *Celastrina lucia* (h01) and *Celastrina neglecta* (h02, h04). Voucher data is given in Suppl. material 1.

DNA barcode data were available for three North American *Celastrina* species (*Celastrina echo*, *Celastrina neglecta*, and *Celastrina lucia*, based on independent identification), and representative Eurasian *Celastrina argiolus* from seven countries (Fig. 2). Eastern Ontario specimens initially identified as *Celastrina lucia*, *Celastrina neglecta* and *Celastrina serotina* are all considered to represent *Celastrina lucia* based on the larval rearing, adult phenology and wing pattern, as discussed below.

Nearly all samples of *Celastrina lucia*, *Celastrina neglecta* and *Celastrina echo* shared an identical DNA barcode. A single haplotype (h03, Fig. 2) was dominant across the continent, representing 76 of 79 individuals and occurring in all three species. Three additional haplotypes (h01, h02, h04; Fig. 2) differed by only a single base-pair, *i.e.* 0.15% divergence, and were represented by a single individual each (Fig. 2). Comparison of these four North American *Celastrina* haplotypes to others in the BOLD database using the sequence identification search engine showed that the extremely conserved genetic variation was not a sampling artefact, with virtually no variation in samples from across North America including Mexico, and including samples identified as all nine North American species in addition to the Mexican *Celastrina gozora* (Bdvl.). This lack of mtDNA genetic differentiation between distinct species occurs also in other North American blues, as a result of introgressive hybridization and possibly infection by the endoparasitic bacterium *Wolbachia* (Gompert et al. 2008). Further research with other molecular markers is needed in *Celastrina*. Although the DNA barcode sequence is not taxonomically informative for North American species, it does corroborate separate species status of *Celastrina argiolus*, which differed by a minimum of 1.4% (mean 1.9%).

### Larval development and host plants

Eggs and larvae of *Celastrina lucia* were found on flower buds and inflorescences of nine species of shrubs in eastern Ontario (Table 3). Based on correlative adult abundance, plant community composition and frequency of larvae on these hosts, *Prunus serotina*, *Cornus alternifolia* L. *Cornus rugosa* Lam., *Viburnum cassinoides* L. and *Viburnum lentago* L. are the most commonly used larval host plants of spring *Celastrina lucia* in this region. *Prunus pensylvanica* L., *Prunus nigra* Aiton and *Amelanchier* species were also searched, but these shrubs bloom very early in the spring with flowers already senescing during peak *Celastrina* abundance, and no larvae were found (Table 3). *Viburnum rafinesquianum* Schult. is rarely used, possibly also due to the later flowering phenology; only one larva was found in searches of 20 shrubs at two different sites (#7, 6; Table 2). Three mature larvae were found feeding on flower buds of *Celastrus scandens* L. in open limestone alvar habitat (site #6). This is the first record of *Celastrus* as a host of *Celastrina*, and adds the family Celastraceae to the list of known host plants (Scott 1986).

**Table 3.** Flowering phenology of deciduous shrubs and larval hosts of *Celastrina lucia* in the Ottawa region.

Phenology	Host^1^	Shrub species	Family	Source^2^	Site #
very early spring	N	*Prunus nigra*	Rosaceae	a	2
	N	*Prunus pennsylvanica*	Rosaceae	a	1,2,5
	N	*Amelanchier* spp.	Rosaceae	a	1,2,4,5,8
	N	*Vaccinium* sp.	Ericaceae	a	1,5
early spring	Y	*Prunus serotina*	Rosaceae	a,b	1,2
	Y	*Prunus virginiana*	Rosaceae	a,b	1,2,8
	(Y)	*Cornus sericea*	Cornaceae	c	-
mid to late spring	Y	*Cornus alternifolia*	Cornaceae	a,b	1,3,4
	Y	*Cornus rugosa*	Cornaceae	a	5
	Y	*Viburnum cassinoides*	Caprifoliaceae	a	1,3,4
	Y	*Viburnum lentago*	Caprifoliaceae	a	1,3,4
	(Y)	*Viburnum rafinesquianum*	Caprifoliaceae	a	6,7
	(Y)	*Diervilla lonicera*	Caprifoliaceae	a	5
	(Y)	*Celastrus scandens*	Celastraceae	a	6
mid summer	Y	*Spiraea alba*	Rosaceae	b	-
(2^nd^ generation)	N	*Spiraea latifolia*	Rosaceae	b	-

1 **N** = Not used as a host; **Y** = Commonly used host; **(Y)** = locally or uncommonly used as a host.

2 **a** = this study; **b** = Layberry (2004); **c** = Eberlie (1998).

Other deciduous shrubs flowering during and after the spring flight season of *Celastrina* were sampled opportunistically, but failed to yield larvae, even when larvae were common on other shrub species at the same sites. These included *Ilex verticillata* (L.) A. Gray, *Ilex mucronata* (L.) (both Aquifoliaceae), and *Lonicera tatarica* L. (an introduced invasive shrub), *Cornus racemosa* Lam., *Vaccinium angustifolium* Ait., and *Gaylussacia baccata* (Wangenh.) K. Koch. An extensive search of the introduced *Viburnum lantana* L. at one site (#2) yielded one half-grown larva, which died several days later in captivity feeding on this plant. *Ilex* is the sole host of *Celastrina idella* Wright and Pavulaan, but is thought to be toxic to *Celastrina lucia* (Wright and Pavulaan 2005). *Cornus sericea* L. is a common host of boreal *Celastrina lucia* populations, but searches for larvae in the study area (site #1) were unsuccessful, despite the patchy but common occurrence of this shrub. Virtually all of the host plants recorded above have completed flowering prior to the onset of summer *Celastrina lucia* flights, which strongly favor *Spiraea alba* Du Roi as oviposition sites and larval hosts (Table 3).

Most of the *Celastrina lucia* host shrubs present in the eastern deciduous forest are absent in the boreal region further north. Within the host genera *Cornus* and *Viburnum*, *Cornus sericea* and *Viburnum edule* (Michx.) Raf. occur widely in the boreal region, but only sporadically in certain plant communities. By contrast, species of Ericaceae are ubiquitous and constitute the main larval hosts in many parts of the ecoregion, particularly plant communities on acidic substrates such as granite barrens, sand plains and bogs. Host plants documented along the James Bay highway in northern Québec in June 2015 (BCS, unpubl. data) included *Cornus sericea*, *Rhododendron groenlandicum* (Oeder) Kron & Judd and *Kalmia polifolia* Wangenh. Searches on *Viburnum edule* and *Prunus pensylvanica* failed to yield eggs or larvae.

**Figure 3.**
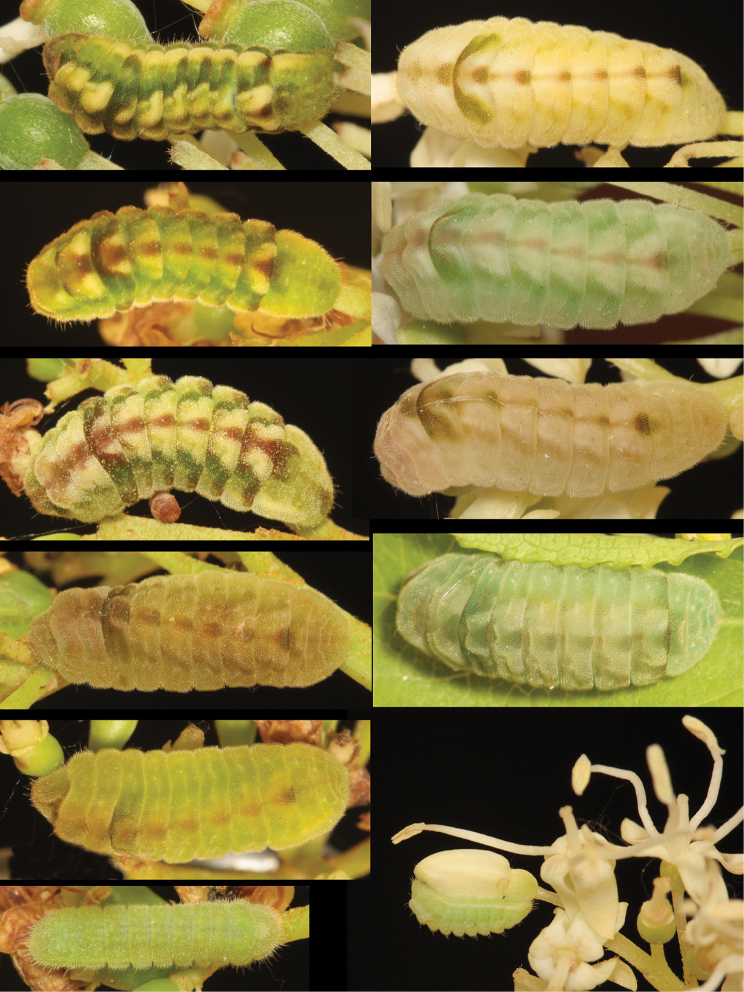
Variation in larval colour pattern of *Celastrina lucia* found on *Viburnum lentago* (Left column) and *Cornus alternifolia* (right column) at site #3 (Table 2).

Larvae found on different plant genera exhibited different colour morph frequencies. Larvae on *Cornus alternifolia* were mostly very pale, pastel-green with little patterning (Fig. 3, right), compared to those on *Viburnum lentago*, which were darker green and more patterned (Fig. 3, left). Three larvae from *Celastrus* flowers were very dark green with little patterning. Differences in larval colours and pattern may represent hostplant-induced variation, not previously documented in *Celastrina* but known to occur in other Lepidoptera (e.g. Sandre et al. 2013).

Of approximately 120 gall-feeding larvae found on 18 *Prunus serotina* trees heavily infested with eriophyid galls, 28 were retained for rearing. Based on size and duration to pupation, approximately 75% were penultimate or ultimate instar, but younger instars were present also. A similar age distribution was observed among *Celastrina* larvae on other hosts at the same time and location, based on collection of 30 larvae from *Viburnum lentago* and *Cornus alternifolia*. Twenty-two of 28 larvae from galls survived to pupation, with five adults emerging between June 29th and July 2^nd^ (summer phenotype) and three more emerging within 9 days at room temperature (spring phenotype) after a 95-day treatment of winter diapause conditions at 5 °C in a conventional refrigerator. The remaining 14 pupae failed to merge and were dissected, revealing fully developed but desiccated adults, which could be assigned to either summer or spring phenotype by comparison to pinned specimens (Fig. 10). In total, 13/22 (59%) and 9/22 (41%) individuals displayed spring versus summer phenotype.

Similar results were obtained from rearing of cherry-gall feeding larvae collected in June of 2004 (RAL), where some pupae yielded summer-phenotype adults in the same year, and some entered diapause to emerge as spring-phenotype adults the following year (Suppl. material 1).

Phenology of cherry gall-feeding larvae was not notably different from that of larvae on other hosts, contrary to the prediction that larvae should appear later based on a later flight period in late May to late June, after that of *Celastrina lucia* (Pavulaan and Wright 2005). Mature larvae found on 11^th^ June would have to be derived from adults flying at least three weeks earlier, assuming 5d for egg hatch and 16d for larval growth even under constant temperatures of 19 °C or more (Table 4). No small larvae were present after June 20^th^. The gall-feeding larvae showed the same size/age distribution as *Celastrina lucia* larvae collected from *Viburnum* and *Cornus*. Neither the larval phenology nor the summer-emerging adults resulting from gall-feeding spring larvae support that gall-feeding larvae represent a separate species, i.e. *Celastrina serotina*. Furthermore, both May and August larvae, initially thought to represent *Celastrina lucia* and *Celastrina neglecta* (Suppl. material 1), can yield either summer adults from non-diapausing pupae or spring adults from diapausing pupae.

**Table 4.** Life cycle duration of non-diapausing *Celastrina lucia*.

Stage	Duration (days)				Temp. (deg. C)	Source region	Data source
	min	max	avg	*n*			
**egg**	3	6	4.5	-	19–22	Washington	James and Nunnallee (2011)
**Larva**	12	22	16.4	*5*	21	Ontario	This study
	16	25	20.5	-	18–27	Washington	James and Nunnallee (2011)
**Pupa**	11	14	12.7	*7*	21	Ontario	Layberry (2004)
	8	19	13	*5*	21	Ontario	This study
	7	13	10	-	18–27	Washington	James and Nunnallee (2011)
	7	-	-	-	22	Michigan	Wagner and Mellichamp (1978)

The alternative taxonomic explanation is that gall-feeding larvae are *Celastrina lucia*, utilizing an unusual plant resource that is, however, similar to a *Prunus* flower bud in size, shape, tissue consistency, and likely phytochemistry. With a relatively long spring flight period and short flowering phenology for a given host species, *Celastrina lucia* must use a suite of hosts to match larval development to host phenology. Galls extend the temporal availability of *Prunus* as they are present longer than flower buds. The total flight season for *Celastrina lucia* is over a month in a given year (Table 6), yet any particular hostplant provides optimal forage for a considerably shorter period. For example *Prunus virginiana* is one of the first hosts to have flower buds, but once flowering begins, females avoid them in favour of other host species.

### Degree days

Comparing degree-day accumulation to flight abundances provides a standardized comparison of flight seasons between southern and eastern Ontario (as defined here), where different climatic conditions prevail. In other words, peak adult emergence is expected to have similar degree-day (DD_6_) accumulation values (dictated by physiological developmental constraints) in regions with differing climates, even though flight times could have quite different calendar dates. Furthermore, DD_6_ accumulation can be used to assess if climatic conditions are amenable to producing multiple yearly generations (multivoltinism).

**Figure 4.**
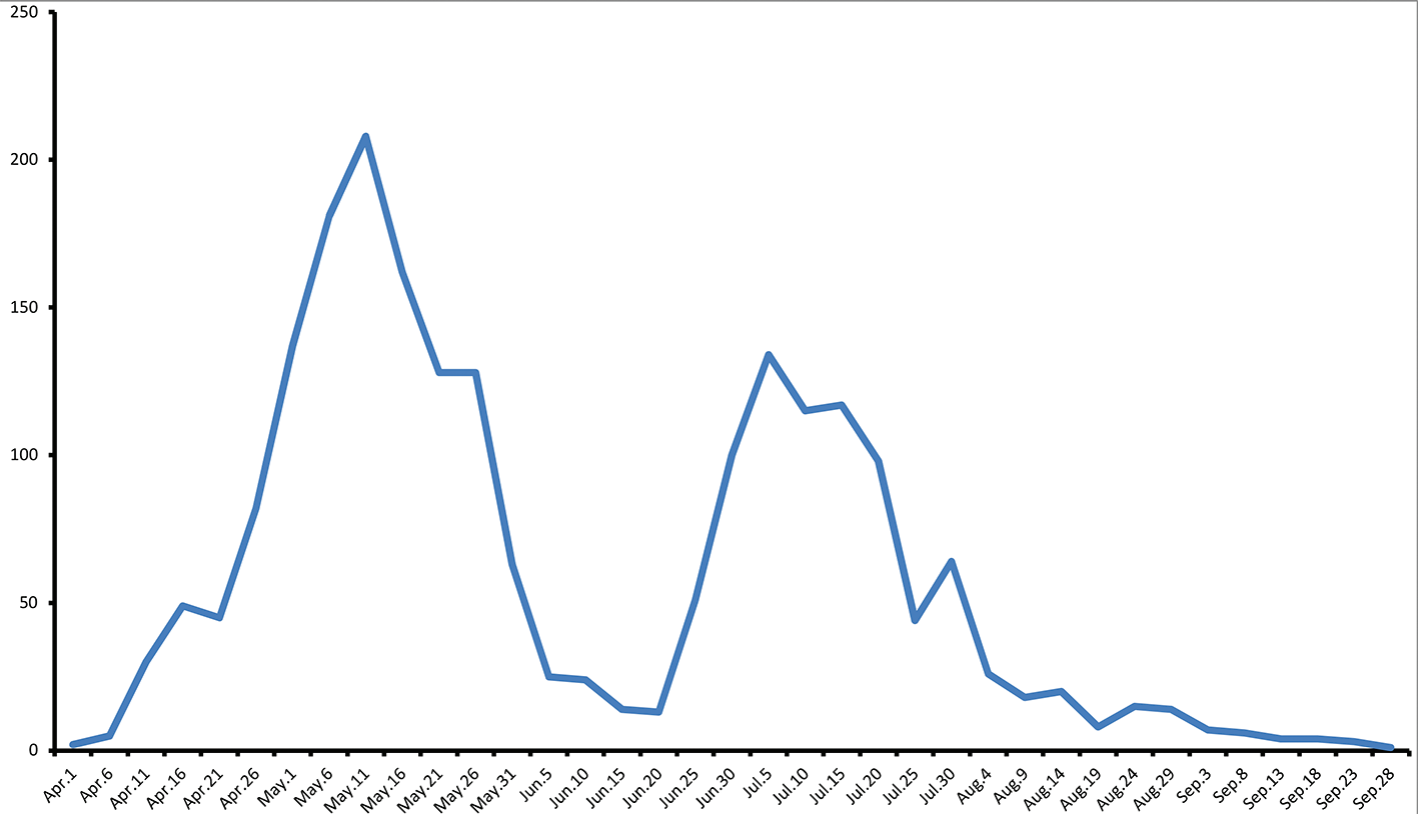
Frequency plot of *Celastrina* adults for eastern Ontario based on cumulative observations from 1899–2014 (n = 2145).

**Figure 5.**
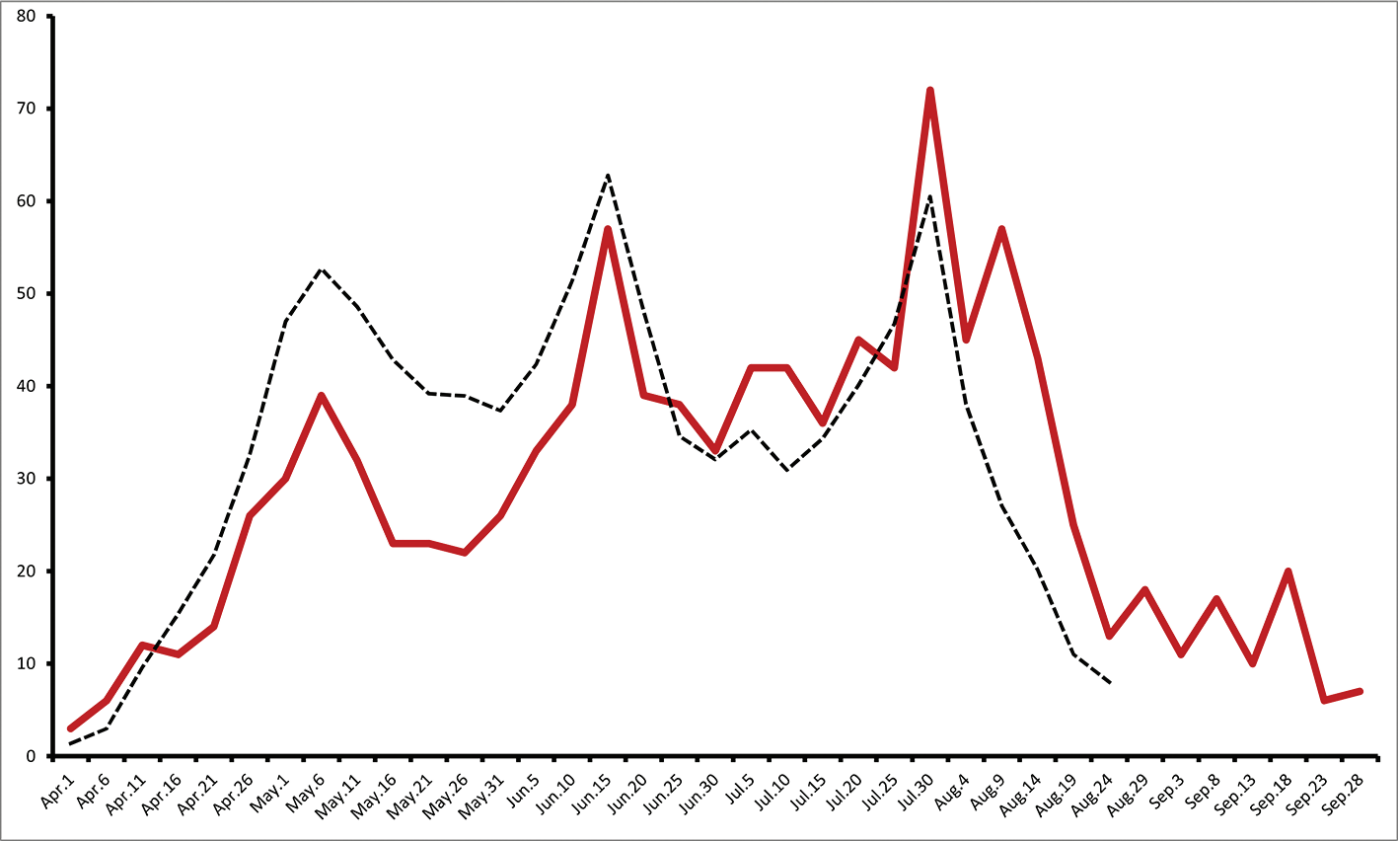
Frequency plot of *Celastrina* adults for southern Ontario (red line) based on cumulative observations from 1895–2014 (n = 1056). Dashed line represents abundance of all *Celastrina* observations assuming hypothetical phenology given in Figure 6.

Cumulative DD_6_ during the spring and summer months was greater for southern compared to eastern Ontario (Table 5). In mid- to late April, DD_6_ accumulation in eastern Ontario lags behind that of southern Ontario by 4–6 days. As the season progresses, the time lag between the two regions diminishes to 2–3 days, for May to the end of July (Table 5). The faster DD_6_ accumulation in southern Ontario is correlated with a slightly earlier spring *Celastrina* peak in that region, occurring on average three days earlier (Figs 4, 5). Large differences between abundance peaks (more than one week) observed between regions are therefore not likely attributable to regional variation in development times of the same species, assuming similar development rates and thresholds between regions.

**Table 5.** Comparison of accumulated degree-days (DD_6_) on selected dates for Ottawa and London, Ontario, based on daily temperatures averaged for 2009–2015. Time lag represents the number of days that London is ahead of Ottawa, based on DD_6_ values averaged for the preceding week.

Date	Ottawa	London	Time lag (d)
01-Apr	0.0	0.0	0.0
10-Apr	0.7	3.4	5.5
20-Apr	10.5	21.5	5.5
30-Apr	35.2	51.4	4.3
10-May	105.3	123.4	2.5
20-May	179.8	200.5	2.5
30-May	288.2	314.4	2.1
10-Jun	403.0	431.2	2.5
20-Jun	528.0	562.6	2.5
30-Jun	675.7	707.0	2.2
10-Jul	830.5	854.7	1.6
20-Jul	987.1	1011.5	1.5
30-Jul	1132.8	1158.3	1.8

**Table 6.** Phenology of *Celastrina* in the Ottawa region April–July 2015.

**Date**	**adults^1^**	**eggs**	**larvae**	**pupae**	**Note**
Apr 19	**X**	|	|	|	First-of-year (FOY) record for adults; only males present
Apr 28	**X**	|	|	|	adults common, FOY females
May 6	**X**	**x**	|	|	adults common, female oviposition behaviour observed
May 12	**X**	**X**	**X**	|	Hatched and unhatched eggs at site #2
May 14	**X**	**X**	**X**	|	Adults, eggs, and larvae at site #1
May 21	**x**	**X**	**X**		Eggs and larvae present but no adults (site #5)
May 26	**X**	**x**	**X**	|	Adults and mature larvae (site #1)
May 28	**x**	**x**	**X**	|	Mature larvae (site #1)
May 29	**X**	**x**	**x**	|	End of flight period, only 3 worn adults seen in 3h
Jun 2	**X**	**x**	**X**	|	One worn adult
Jun 4	**X**	**x**	**X**	|	One worn adult
Jun 9	|	|	**x**	**x**	FOY pupae predicted^2^
Jun 11	|	|	**X**	**x**	Larvae (site #3,4)
Jun 14	|	|	**X**	**x**	Larvae (site #3)
Jun 18	|	|	**X**	**x**	Larvae (site #3)
Jun 20	**X**	|	|	**x**	FOY summer brood adults - male

1 **X** = presence based on direct observation; **x** = presence inferred based on observation of another life stage; | = absent

2 No pupae were found in the field. Presence of pupae is predicted based on degree-day values for a larval stage duration of 17d at 21C (Table 4), subsequent to first observed larval presence on May 12th.

Is it possible that summer abundance peaks represent the offspring of spring *Celastrina*? Currently, spring and summer *Celastrina* are treated as separate species, and some have maintained that *Celastrina* flying subsequent to the spring flight appear too soon for this to be possible (e.g. Saunders 1875). In eastern Ontario, the median abundance dates occur on May 11^th^ and July 12^th^ (Fig. 4; 50% of observations for the period prior to June 12st or after June 20^th^). In southern Ontario, however, the situation is different, as there is an abundance peak with a mean date of June 15^th^, after a spring peak on May 8^th^. The time lag between the first two seasonal peaks is therefore between May 11^th^ - July 12^th^ in Eastern Ontario and May 8^th^ - June 15^th^ in southern Ontario, corresponding to an average degree-day (DD_6_) of 750 and 381, respectively (data not shown; degree-day trends in Table 5). These DD_6_ values likely represent a slight overestimate of actual degree-days available for completion of a generation, since the between-peak time lag does not account for the fact that most eggs are probably laid after the peak flight period. This is due to females emerging later than males and being less commonly observed, as is true for nearly all butterflies (Scott 1986).

Average life cycle duration of non-diapausing *Celastrina* in Ontario (35d total; egg = 5d, larva = 17d, pupa = 13d at 22 °C; Table 4), has an accumulated DD_6_ value of approximately 560, considerably greater than the maximum estimated DD_6_ of 381 available in southern Ontario, but less than the DD_6_ of 750 in eastern Ontario. Degree-day modelling data therefore indicates that there are enough degree-days between the first and second abundance peaks to permit development of a second generation in eastern Ontario but not in southern Ontario, and the two peaks in the latter region cannot therefore represent the same species.

### Adult phenology

*Celastrina* phenology in eastern Ontario exhibits a bimodal pattern, with a well-defined spring and summer peak. Median spring abundance (*i.e.*, 50% of records) occurs on May 8^th^ and median summer abundance on July 12^th^ (spring and summer periods divided by the trough midpoint at June 21^st^). *Celastrina* abundance drops sharply between June 5^th^ and June 24^th^; in other words, azures of any kind are very rarely observed in eastern Ontario during this period (Fig. 4). This is opposite to the pattern seen in southern Ontario, where a June 15–19^th^ abundance peak occurs in addition to a May and July/August peak (Fig. 5).

Another notable difference in *Celastrina* phenology between eastern versus southern Ontario is the magnitude of spring (April–May) versus summer (July onwards) abundance peaks. In southern Ontario, there are considerably fewer spring than summer records, the converse of the pattern in eastern Ontario. *Celastrina* abundance also persists further into the summer in southern Ontario, not declining significantly until after Aug 21^st^, compared to steady declines after mid-July in eastern Ontario (Fig. 5).

The bimodal abundance pattern in eastern Ontario reflects at minimum two entities, a spring- and a summer-flying *Celastrina*, previously considered to be *Celastrina lucia* and *Celastrina neglecta*, respectively. The time lag between spring and summer emergences, and the rearing results and phenotype comparisons discussed below, indicate that eastern Ontario spring and summer *Celastrina* represent two broods of the same species, *Celastrina lucia*.

Although there is no evidence of a third peak (in eastern Ontario) intercalated between the first and second as would be expected for *Celastrina serotina*, it is possible that such an abundance signature is hidden by virtue of *Celastrina serotina* being much rarer than *Celastrina lucia* and *Celastrina neglecta*. However, the 2015 observations on larval and adult phenology do not support this (Table 6). No “flush” of freshly emerging adults appeared after the peak of *Celastrina lucia* adults, and there was no detectable difference in age (size) of cherry-gall feeding larvae compared to other larvae. What, then, is the true identity of *Celastrina* previously attributed to *Celastrina serotina*? To address this, all eButterfly (2015) *Celastrina serotina* records with voucher photographs were examined, consisting of 28 records with dates ranging from 14 May to 26 June. Both worn *lucia*-like individuals and freshly emerged *neglecta*-like individuals are identified as *serotina*, the primary means of identification apparently being date. Fourteen individuals were visually indistinguishable from either worn individuals of *Celastrina lucia* or fresh, lightly marked (form “violacea”) individuals thereof. Ten individuals were fresh with a chalky–white ventrum and small, sharp spots and little to no marginal markings, like those of the June-flying, southern Ontario entity here assigned to *Celastrina neglecta*. Specimens identified as *Celastrina neglecta* tended to occur further south than *Celastrina lucia* (Fig. 8).

**Figure 6.**
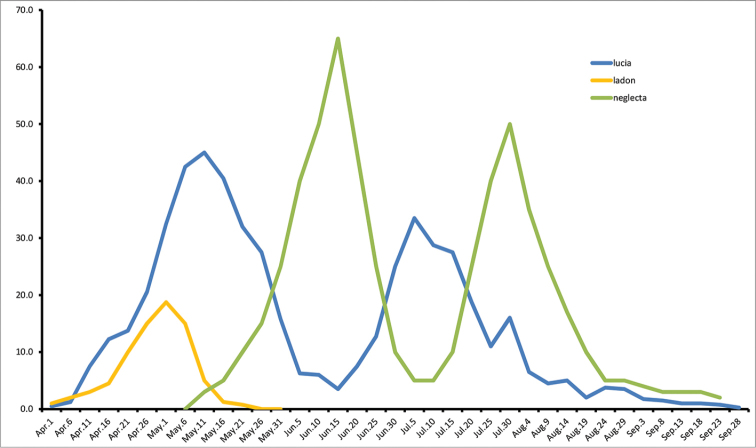
Hypothetical phenology of *Celastrina* species in southern Ontario. *Celastrina lucia* abundance is based on eastern Ontario data (Figure 4), *Celastrina neglecta* data is based on assumption of two annual flights, the first peaking in mid-June and with a generation time similar to that of Celastrina lucia (750 degree-days). *Celastrina ladon* data is based on assumption of a single, earlier flight and lower overall abundance compared to *Celastrina lucia*, but with similar abundance changes and length of flight period. The sum of all predicted *Celastrina* abundances is compared to actual observation frequencies in Figure 5.

**Figure 7.**
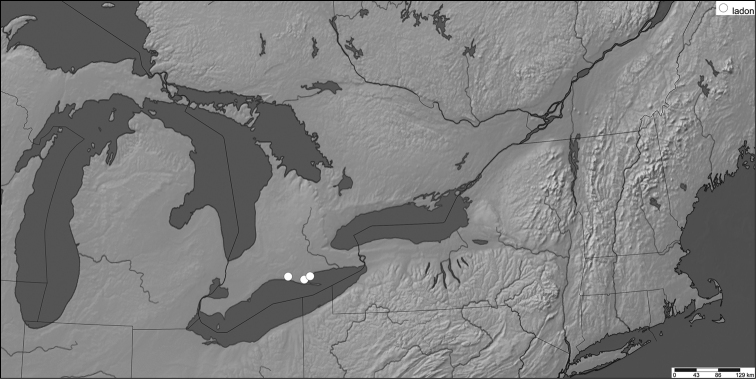
Distribution of examined voucher specimens for *Celastrina ladon* in Ontario.

**Figure 8.**
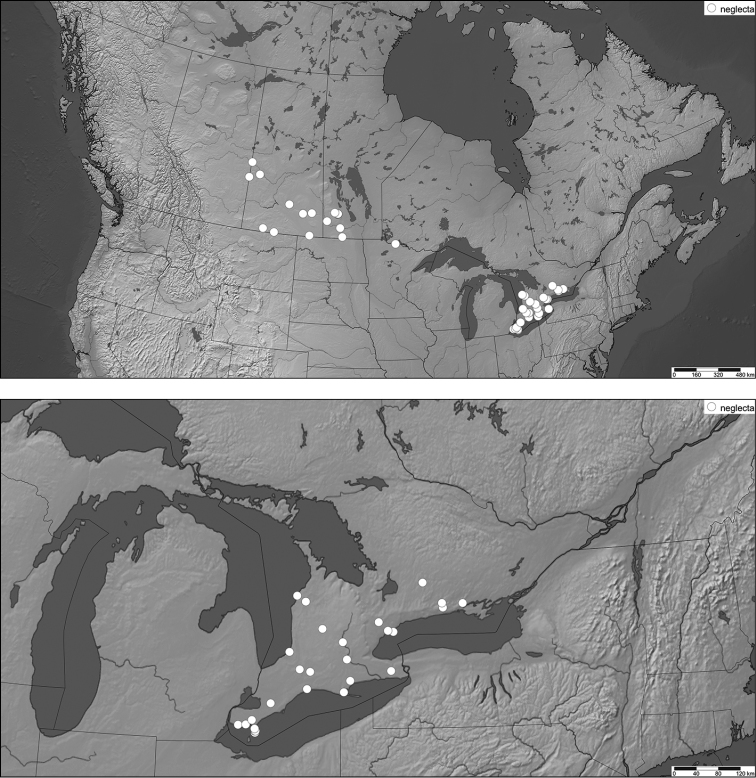
Distribution of examined voucher specimens for *Celastrina neglecta* in Canada (above) and Ontario (below).

**Figure 9.**
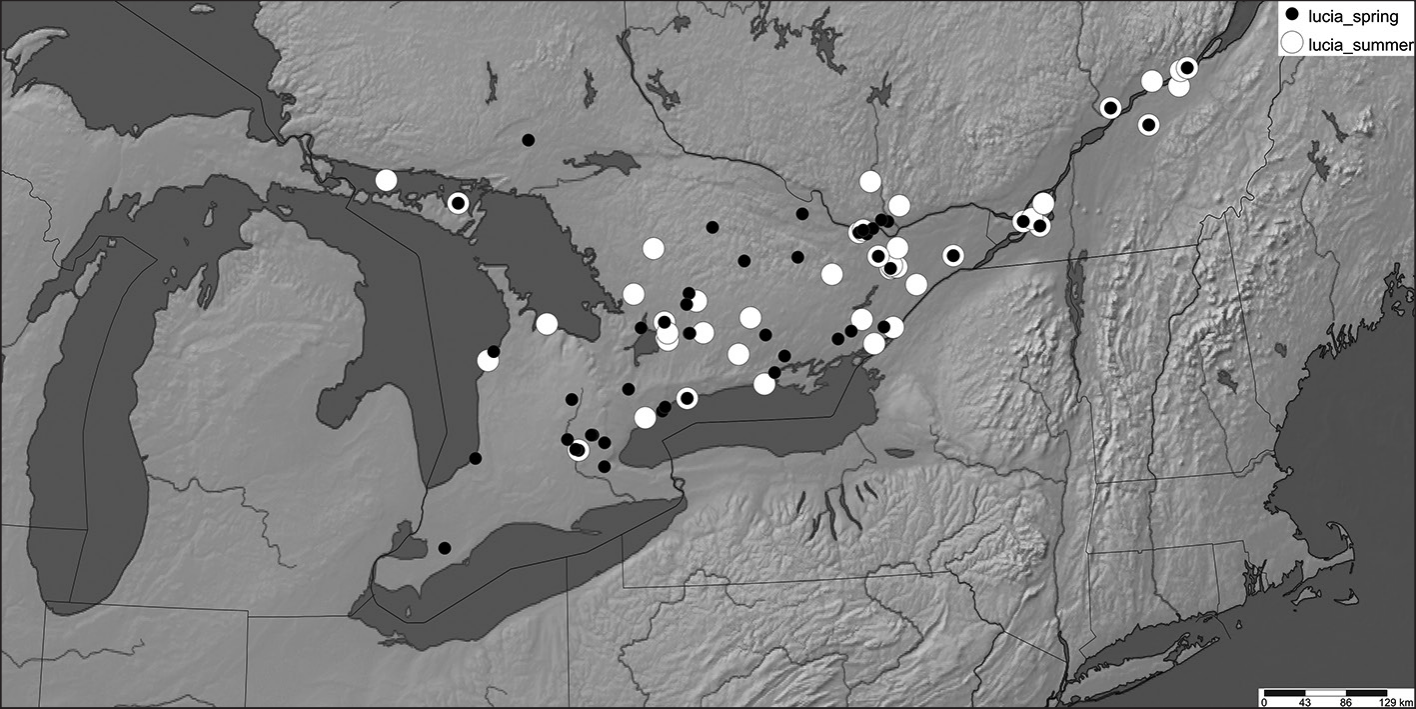
Distribution of examined voucher specimens for spring (black circles) and summer (white circles) *Celastrina lucia* in southern Ontario and adjacent Québec.

**Figure 10.**
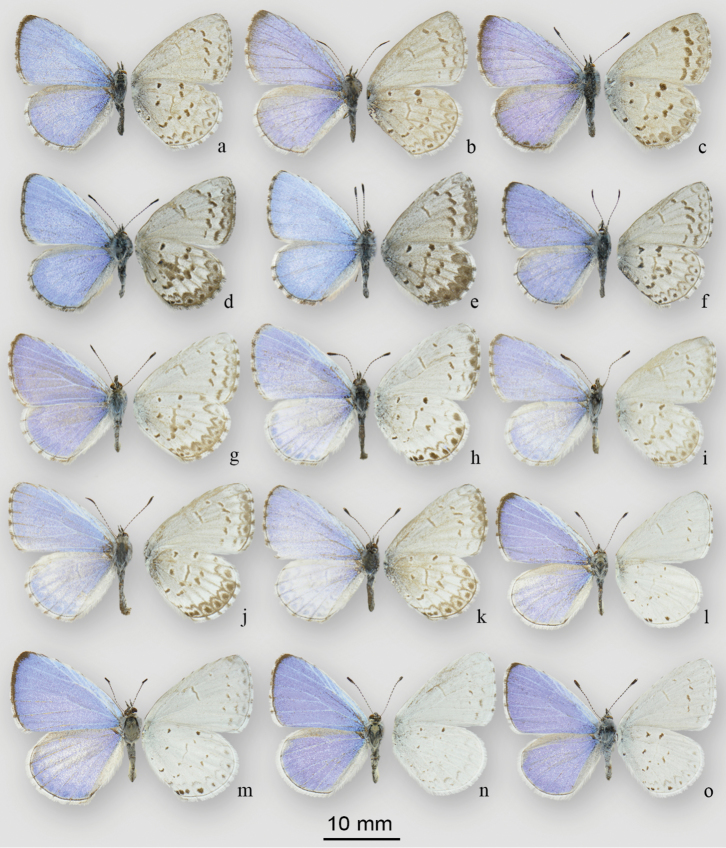
Adult males of *Celastrina*. **a–c** *Celastrina ladon* (Cramer) **a** Normandale, ON, CAN, 22 May 1956, J.R. Lonsway, (CNCLEP 116459) **b** St Williams, ON, CAN, 7 May 1977, J.T. Troubridge (CNCLEP 116460) **c** St Louis, Missouri, United States, 15 April 1979 (CNCLEP 116461) **d–f** *Celastrina lucia* (Kirby), spring generation **d, e, f** Stony Swamp, Richmond Road, Ottawa-Carleton, ON, 45.298°N, 75.828°W, CAN, 28 April 2015, B.Celastrina Schmidt (CNCLEP 116445, 116447, 116446) **g–k** *Celastrina lucia* (Kirby), summer generation **g** Riding Mtns., MB, 12 June 1938, J. H. McDunnough (CNCLEP 116448) **h** Timm Dr., Ottawa, ON, 45.315°N, 75.860°W, CAN, 14 May 2015, B.Celastrina Schmidt (CNCLEP 116451) **i** Bobcaygeon, ON, CAN, 16 July, 1932, J. McDunnough (CNCLEP 116453) **j** Pont Neuf, QC, CAN, 8 July 1973, no collector (CNCLEP 116454) **k** Britannia, Ottawa, ON, CAN, 30 June 1949, R. deRuette (CNCLEP 116455) **l–o** *Celastrina neglecta* (Kirby) **l** Larsson’s Camp, One Sided Lake, ON, CAN, 19 June 1960, M.R. MacKay (CNCLEP 116464) **m** Point Erie, ON, CAN, 6 August 1950, T.N. Freeman (CNCLEP 116465). Riding Mountains, MB, CAN, 13 June 1938, J. McDunnough (CNCLEP 116466) **o** Riding Mountains, MB, CAN, 12 June 1938, J. McDunnough (CNCLEP 116467).

In southern Ontario, spring *Celastrina* are rare compared to the abundance of azures seen from June onwards (Layberry 1996). Saunders (1875) noted that *Celastrina* were absent prior to late May in the London area. This pattern is reflected by fewer spring vs. summer observations, and the presence of an additional June flight peak that is absent in eastern Ontario. Comparison of June *Celastrina* from southern Ontario to those from other areas reveals that these also differ phenotypically (Figs 9, 10). As discussed under *Celastrina neglecta* in the Conclusions section, this taxon is here deemed to be *Celastrina neglecta*.

The spring/summer abundance discrepancy in southern Ontario was also noted by Layberry (1996), who stated that spring *Celastrina* were rare and local and could not possibly produce the abundance of ubiquitous summer *Celastrina*. This discrepancy can be explained by the localized occurrence of *Celastrina lucia* (near its southern range limit) and *Celastrina ladon* (restricted to Carolinian woods) in spring, followed by the much more common *Celastrina neglecta* in late May–June and again in Late July–August.

The complex abundance peaks for southern Ontario are at least in part a result of combined data for multiple species. Degree-day modelling can however be used to approximate the apparent abundance peaks. Given a spring peak of *Celastrina lucia* on May 8, and an average DD_6_ accumulation of 750 to reach the second-brood peak (based on the eastern Ontario phenology), summer *Celastrina lucia* would be expected to peak on July 11^th^ on average. A corresponding, although weak, peak occurs in southern Ontario between July 5^th^ and 14^th^ (Fig. 5). Assuming similar physiological development rates and parameters for *Celastrina neglecta*, a summer peak of 750 DD_6_ after the June 15th peak would be expected, corresponding to August 5^th^. This correlates well with the observed peak between July 30^th^ and Aug 3^rd^ (Fig. 5).

### Identification and distribution of Canadian *Celastrina neglecta*

To establish comparative phenotypes of *Celastrina neglecta* and summer-brood *Celastrina lucia*, southern Ontario specimens collected during the June flight peak (Figure 5) were compared to July specimens from eastern Ontario (summer peak; Figure 4). This provided a conservative estimate of phenotypic variation in *Celastrina neglecta*, which differs in having darker, smaller and more sharply defined ventral spots, brighter white ventral ground colour, more reduced marginal markings, a solid white dorsal hindwing fringe, and more pronounced dark marginal shading of the forewing apex (compare Fig. 10g–k to 10l–o). To define the distribution of *Celastrina neglecta*, a conservative approach was taken to avoid construing summer *Celastrina lucia* with first or second generation *Celastrina neglecta*. For Ontario and Québec, specimens were identified as *Celastrina neglecta* only if they met two criteria, *i.e.* matching the *Celastrina neglecta* phenotype as above, and a collection date between late May and late June, prior to the onset of the summer *Celastrina lucia* flight. For the Prairies and Maritimes region where flight period is expected to be later compared to Ontario, all available specimens previously identified as *Celastrina neglecta* were evaluated. Specimens previously identified as *Celastrina neglecta* from all parts of the Canadian range revealed that true *Celastrina neglecta* occurs from easternmost Alberta to southern Ontario. Specimens from eastern Ontario, Québec and the Atlantic region match the summer *Celastrina lucia* phenotype, consistent with the notion that Maritimes *Celastrina* all represent a single, partially bivoltine species (Maritimes Butterfly Atlas 2015). Two Nova Scotia specimens reared from *Aralia* (CNC) previously identified as *Celastrina serotina* (Pavulaan and Wright 2005) were also re-identified as summer brood *Celastrina lucia*.

In Canada, *Celastrina neglecta* is sympatric with *Celastrina lucia* in nearly all parts of the *neglecta* range. Most summer records from the Prairie Provinces proved to be *Celastrina neglecta* (Fig. 8), although summer brood *Celastrina lucia* occur also in southern Manitoba (Fig. 10), and are expected in Saskatchewan based on a single recent record from as far west as Edmonton, Alberta. In Ontario, *Celastrina neglecta* has a more restricted southern distribution compared to bivoltine *Celastrina lucia* populations, so far documented to about 44 °N (Fig. 8b). The maximum northeastern extent is currently at the eastern edge of the Oak Ridges Moraine (Rice Lake Plains) and the southern Napanee Limestone Plain (Fig. 8b). In southern Ontario it is the most common *Celastrina*, and both *Celastrina lucia* (Fig. 9) and *Celastrina ladon* (Fig. 7) have a more localized occurrence. Field work is needed to definitively establish the northern range limit, especially in the regions of Georgian Bay, Bruce Peninsula, and the Frontenac Arch. No Québec vouchers were located but the species could be expected in regions know for southern species, such as the southern Richelieu River valley and the Lake Champlain region.

## Conclusions

The Canadian *Celastrina* fauna is revised to consist of four species: *Celastrina lucia* (all provinces and territories), *Celastrina neglecta* (southern Ontario to eastern Alberta), *Celastrina ladon* (Carolinian zone of southernmost Ontario), and *Celastrina echo* (southern British Columbia and southwestern Alberta). From eastern Ontario eastward, what was previously treated as three *Celastrina* species is revised to a single, facultative bivoltine species, *Celastrina lucia*. Adults of *Celastrina lucia* flying from early to mid-spring, in a relatively prolonged emergence, give rise to a second and possibly a partial third generation in July to September. Larval rearing, phenology, and seasonal emergence patterns show no evidence of *Celastrina serotina* as a separate gall-feeding species distinct from *Celastrina lucia*, and *Celastrina serotina* is therefore removed from the Canadian fauna. Whether or not nominate *Celastrina serotina* (described from Rhode Island) is a valid species, or simply represents late-emerging *Celastrina lucia* that utilize cherry galls, needs to be re-evaluated. Molecular markers such as microsatellites could prove to be particularly valuable in advancing the taxonomy of *Celastrina*, given that the COI barcode marker is taxonomically uninformative here.

### *Celastrina lucia*

**Figure 11.**
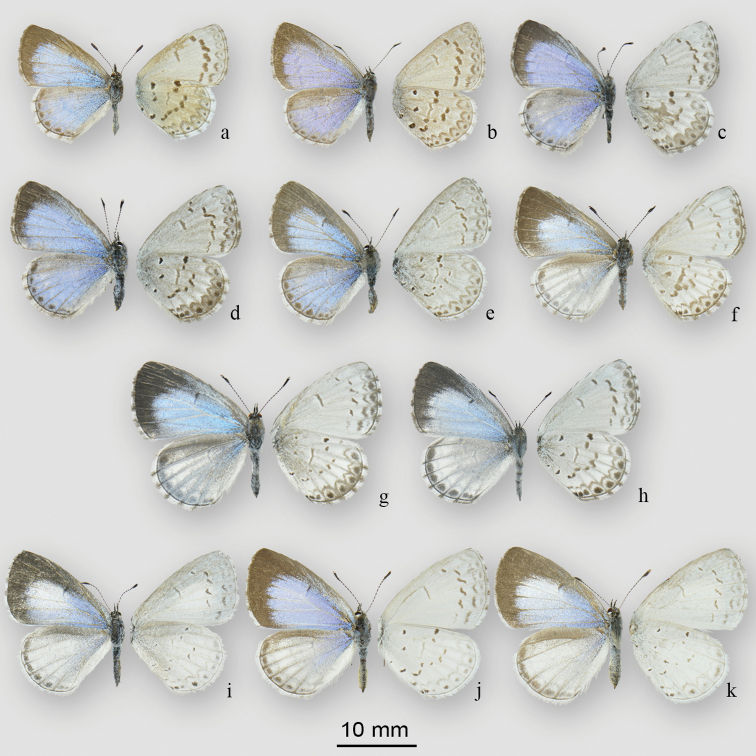
Adult females of *Celastrina*. **a–b** *Celastrina ladon* (Cramer) **a** Normandale, ON, CAN, 28 May 1956, J.R. Lonsway (CNCLEP 116462) **b** Lake Wellington, Washington Co, Arkansas, United States, 12 April 1974 no collector (CNCLEP 116463) **c–e** *Celastrina lucia* (Kirby), spring generation **c** Stony Swamp, Richmond Road, Ottawa-Carleton, ON, 45.298°N, 75.828°W, CAN, 28 April 2015, B.Celastrina Schmidt (CNCLEP 116449) **d** Bells Corners, Timm Road, Ottawa, ON, 45.315°N, 75.860°W, CAN, 14 May 2015, B.Celastrina Schmidt (CNCLEP 116450). **e)** Timm Dr., Ottawa, ON, 45.315°N, 75.860°W, CAN, 14 May 2015, B.Celastrina Schmidt (CNCLEP 116451) **f–g** *Celastrina lucia* (Kirby), summer generation **f** Château-d’Eau, QC, CAN, 21 July 1990, J.-P. Laplante (CNCLEP 116456) **g** *Celastrina lucia* (Kirby): 5kmSE of Fitzroy Harbour, Fitzroy, ON, 45.4348°N, 76.1725°W, CAN, em 20 June 2015, Ross Layberry (CNCLEP 116457) **h** Stony Swamp, Richmond Road, Ottawa-Carleton, ON, 45.297°N, 75.836°W, CAN, 2 July 2015, B.Celastrina Schmidt (CNCLEP 116458) **i–k** *Celastrina neglecta* (Kirby) **i** Harrow, Essex Co., ON, 42.0390°N, 82.9080°W, 28 May 2015, Jeff Larson (CNCLEP 116468) **j** Simcoe, ON, CAN, 26 June 1939, T.N. Freeman (CNCLEP 116469) **k** Bobcaygeon, ON, CAN, 22 June 1932, J. McDunnough (CNCLEP 116470).

Two additional possibilities in the identity of the species here assigned to *Celastrina lucia* warrant comment. It is conceivable that *Celastrina neglecta* is present as a univoltine, summer-flying entity that is phenotypically similar to and unrecognized within summer-brood *Celastrina lucia*. This would require that the June-flying *Celastrina* in southern Ontario be *Celastrina serotina*, and that *Celastrina neglecta* in eastern Ontario overwintering as pupae delay emergence until July. Both of these conditions are improbable; the identity of June *Celastrina* in southern Ontario is most likely *Celastrina neglecta* as discussed below, and there are no known temperate-zone Lycaenidae that overwinter as pupae and delay emergence until July. Eastern Ontario summer *Celastrina* also have the appearance of pale *Celastrina lucia* (Figs 10, 11).

The second possibility is that the eastern Ontario taxon represents a species distinct from nominate *Celastrina lucia*, that is *Celastrina lucia* ‘of authors’ in the sense of Pratt et al. (1994), based on larger size, wing pattern differences, and differing host plant preferences. This interpretation remains to be thoroughly evaluated, particularly by examining latitudinal gradients of the character traits in question. For now, we favour the simplest taxonomic hypothesis, where this taxon represents *Celastrina lucia* with facultative bivoltine populations, clinally variable phenotypes and regional host plant preferences.

Although consistently stated to be univoltine in the literature, *Celastrina lucia* is here interpreted to be facultatively bivoltine (and possibly trivoltine) in southern Canada (Fig. 9), with northern, boreal populations being univoltine. In addition to climatic conditions, voltinism may be regulated by host plant availability (Shapiro 1975), explaining why more southerly populations of *Celastrina lucia* could be strictly univoltine (Pavulaan 2014). Plasticity in voltinism is perhaps not surprising given that the Eurasian sister species *Celastrina argiolus*, occupying very similar ecological niches, is also well known to be facultatively bivoltine (e.g. Ebert 1993). *Celastrina echo* is well-known to be bivoltine in western North America, and some western *Celastrina lucia* populations can produce a second generation under laboratory conditions (James and Nunnallee 2011). Similar mechanisms of geoclimatically variable voltinism are common and taxonomically widespread in Lepidoptera, although perhaps less prevalent in temperate butterflies. As *Celastrina* is primarily a tropical group, multivoltinism is likely an ancestral evolutionary trait, with univoltinism a derived trait adaptive for climatic or host plant limitations.

Rearing data indicate that a proportion of spring individuals of Ontario *Celastrina lucia* enter diapause the following spring (Eberlie 1997; Layberry 2004; this study). Summer observations are 45% fewer than in spring (Fig. 4), suggesting that roughly half of the individuals resulting from the spring brood enter diapause. 59% of pupae reared in 2015 similarly did so. Triggers for facultative bivoltinism are in part environmental, as flight phenology shifts later into the spring with latitudinal climatic amelioration. Warmer spring temperatures as a result of climate change are expected to favour northward expansion of bivoltinism in *Celastrina lucia*. This was recently documented in Alberta with the first recorded summer brood *Celastrina lucia* (G. Anweiler, pers. comm; photo examined), in an area with a century of butterfly surveying (Pohl et al. 2009). *Celastrina lucia* therefore provides an excellent opportunity to study the effects of climate change on developmental thresholds.

Larvae of *Celastrina lucia* are polyphagous, but show preferences for several genera in different families (Table 3) and feed almost exclusively on flowers and fruits. *Celastrina lucia* uses a variety of host plants with differing flowering phenologies to span the duration of a relatively lengthy flight period. As part of this dietary strategy, *Celastrina lucia* also feeds opportunistically on leaf galls of *Prunus serotina* and *Prunus virginiana*, which has been documented in Québec, Ontario and Manitoba, but is likely a geographically more widespread phenomenon.

### *Celastrina neglecta*

In southern Ontario, a third *Celastrina* species appears in late spring after an initial May flight of both *Celastrina lucia* and *Celastrina ladon*. The appearance of this species is too soon after the first flight of *Celastrina* to represent a second annual generation. Adult wing phenotype is similar to the summer brood of *Celastrina lucia*, but differs in having darker, smaller and more sharply defined ventral spots, more reduced marginal markings, a solid white dorsal hindwing fringe, and a less evenly checkered forewing fringe (Table 7). The differences between *Celastrina neglecta* and summer *Celastrina lucia* requires more study, and the diagnosis and accompanying figures given here should be treated as a guideline for further research rather than a definitive diagnostic tool.

**Table 7.** Differential diagnosis of Celastrina species in Ontario, Canada.

Species	Maximum annual # generations	Peak flight times	Distribution	Male forewing androconial scales	Male forewing overlapping scales	Ventral hindwing: confluent discal macules	Ventral Hindwing: confluent marginal markings	Ventral hindwing: expression and colour of marginal markings	Ventral ground colour	Hindwing fringe	Dorsal hindwing	Dorsal forewing
*Celastrina lucia*	3?	E - L May; E - L Jul	Ubiquitous through most of province; localized south of 43N	present	absent	common	common	spring: well-developed, diffuse, brown to grey. Summer: moderately developed, usually light brown-grey	Spring - grey to greyish white; rarely white. Summer - white	white with black fringe at vein termini	summer: extensive white scasling; ventral pattern usually visible	Fringe and terminal area evenly checkered from apex to tornus, or slightly darker at apex
*Celastrina neglecta*	2?	L May - L June; L Jul - L Aug	Primarily south of 44.5N, but northern limits uncertain	present	absent	rare	rare	poorly developed, small and dark grey; marginal crescents often absent	white	solid white	limited or sparse white scaling; ventral pattern not usually visible	Fringe and terminal area darker in apical area, with termina lblack line usually widest at apex
*Celastrina ladon*	1	L Apr - M May	Carolinian zone south of 43N	absent	present	rare	rare	moderately developed, diffuse, brown to grey	grey to greyish white	white with black fringe at vein termini	limited or no white scaling	Margin evenly checkered from apex to tornus, or slightly darker at apex

In Ontario, this taxon was recognized as distinct from *Celastrina lucia* 140 years ago by Saunders (1875), who considered it to be the most common *Celastrina* in the London area, appearing in late May to early June. Pavulaan and Wright (2005) assigned Saunders’ records to *Celastrina serotina* (although Saunders (1869) states that specimens were reared from larvae found on *Cornus*). The abundance of this species in the absence of *Prunus serotina* in southern Ontario (R. Cavasin, pers. comm.), and the larval host plant records discussed below, indicate that this species is not *Celastrina serotina*. What name to apply to this taxon is however not straight-forward. The differential diagnosis of *Celastrina serotina* and *Celastrina neglecta* is based primarily on phenology, voltinism, and to some extent on host plant (Pavulaan and Wright 2005). Pavulaan and Wright (2005) state that *neglecta* has a single summer flight after that of *Celastrina serotina* in Canada, but when *Celastrina neglecta* has a spring flight, it is before that of *Celastrina serotina*. The phenology of *Celastrina neglecta* as proposed by Pavulaan and Wright (2005) seems counterintuitive as it states that *Celastrina neglecta* has a summer flight in the north but then adds an earlier, spring flight southward. Other facultatively bivoltine Lepidoptera generally have additional flights later not earlier in the year. *Celastrina neglecta* is more intense blue with more white suffusion dorsally, and a weaker ventral maculation pattern compared to *Celastrina serotina* (Pavulaan and Wright 2005). Of course the name of this species hinges on the identity of the lectotype specimen of *Celastrina neglecta*, which surprisingly has not been considered in detail. Until this situation can be thoroughly reviewed, the identity of the June/August *Celastrina* of southern Ontario is most parsimonious with the current concept of *Celastrina neglecta*. Many southern Ontario specimens are also very similar to the Manitoba taxon *argentata* (Fletcher), which is currently considered a synonym of *Celastrina neglecta* (Pelham 2011). The distribution, similar phenotype and phenology of Great Lakes *Celastrina neglecta* Great Plains *argentata*, together with Colorado *Celastrina humulus* Scott and Wright 1999 certainly suggest that these taxa all represent the same species.

Canadian host plant records that are probably attributable to *Celastrina neglecta* include *Ceanothus americanus* (based on late June larvae from Northumberland Co., Ontario; Eberlie 1997; 1998; ovipositing female, Northumberland Co., R. Cavasin, photo examined); *Cornus amomum* Mill. (late June oviposition and larvae at Point Pelee, J. Cossey, photo examined), and *Cornus drummondii* CelastrinaA. Mey (late June to early July larvae from Essex County, J. Celastrina Lucier, Ontario Butterfly Atlas 2015). Host plants of populations in the prairies are completely unknown; both *Cornus* and *Ceanothus* are sparse or absent where these populations occur.

### *Celastrina ladon*

The Spring Azure, *Celastrina ladon*, is here confirmed as part of the Canadian fauna. It is currently known from only three sites, with the most recent record from 2000. Surveys for this species are urgently needed as the primary larval host, Eastern Flowering Dogwood (*Cornus florida* L.), is endangered in Canada (Environment Canada 2014). This species is experiencing population declines in Ontario caused by dogwood anthracnose fungus, forest succession, habitat loss and herbivory by deer (Environment Canada 2014). Oviposition and suitability of other larval hostplants also needs to be established, as it is possible that *Viburnum* and other *Cornus* may be suitable hosts. Remaining core areas for *Cornus florida* in Ontario include Backus Woods, Wilson Tract, Turkey Point PP, Spooky Hollow Nature Sanctuary (COSEWIC 2007).

### Research needs

Surprisingly, there are still many large gaps in our understanding of *Celastrina* taxonomy and biology. The most urgent need for Canadian *Celastrina* research is vouchered surveys for *Celastrina ladon* in southern Ontario, so that potential conservation needs can be established. Regions where *Celastrina neglecta*, *Celastrina lucia* and/or *Celastrina ladon* occur in sympatry provide an excellent opportunity for comparative study, where time series of vouchers are needed to establish diagnostic as well as habitat and host plant differences. Along similar lines, latitudinal transects of voucher series and host use are needed to examine the transition from southern to boreal *Celastrina lucia*.

Lastly, controlled-environment rearing studies of all taxa would establish plasticity in voltinism and developmental requirements and diapause triggers. The use of degree-day modeling could easily be fine-tuned as a useful comparative tool for *Celastrina* taxa and populations, and to model geographic variation of *Celastrina* emergence. Dearborn and Westwood (2014) used a similar approach to predict emergence of an endangered skipper.

## Supplementary materials

Supplementary material 1 Data for DNA barcode voucher specimens of *Celastrina* Data type: Microsoft Excel Spreadsheet (.xls) This dataset is made available under the Open Database License (http://opendatacommons.org/licenses/odbl/1.0/). The Open Database License (ODbL) is a license agreement intended to allow users to freely share, modify, and use this Dataset while maintaining this same freedom for others, provided that the original source and author(s) are credited.Vitor C. Pacheco da Silva, Mehmet Bora Kaydan, Jean-François Germain, Thibaut Malausa, Marcos Botton
